# Dissecting age-specific genetic architecture of vitiligo through integrative Post-GWAS analysis

**DOI:** 10.3389/fimmu.2026.1783791

**Published:** 2026-05-08

**Authors:** Qingqing Si, Shenglan Wang, Junru Chen, Yuanyu Feng, Ruike Zhao, Dongjie Sun

**Affiliations:** 1 Kunming Medical University, Kunming, Yunnan, China; 2 The First Affiliated Hospital of Kunming Medical University, Kunming, Yunnan, China; 3 Department of Dermatology, The First Affiliated Hospital of Kunming Medical University, Kunming, Yunnan, China

**Keywords:** age stratification, genetic heterogeneity, Mendelian randomization, systems immunology, vitiligo

## Abstract

**Background:**

Vitiligo is a chronic acquired depigmentation disorder with a global prevalence of 0.5-2%. Early-onset (EO) and late-onset (LO) subtypes exhibit marked differences in clinical presentation and disease progression, yet their genetic underpinnings have not been fully elucidated.

**Methods:**

We employed linkage disequilibrium score regression (LDSC) to quantify genetic correlation between EO and LO vitiligo, subsequently applying stratified LDSC to evaluate functional annotation and cell-type-specific heritability enrichment across immune cell populations. Multi-marker analysis of genomic annotation identified subtype-associated genes and assessed their potential biological functions and tissue-specific enrichment patterns. Summary-data-based Mendelian randomization (SMR) identified putative core targets at both transcriptional and translational levels, complemented by colocalization analysis to identify putative core targets. Expression specificity of core targets was assessed using peripheral blood single-cell databases, with spatial mapping performed via the genetically informed spatial mapping of cells for complex traits (gsMap) framework. Exploratory clinical validation employed Quantitative Reverse Transcription PCR (qRT-PCR) and Enzyme-Linked Immunosorbent Assay (ELISA )on patient blood samples.

**Results:**

Genetic correlation analysis revealed incomplete overlap between the two vitiligo subtypes (rg = 0.672-0.787), indicating distinct underlying genetic mechanisms for each phenotype. Stratified heritability analysis showed that LO vitiligo exhibited broader heritability distribution across regulatory elements, whereas EO concentrated in evolutionarily conserved regions. Cell-type enrichment differed significantly: EO involved T cells, dendritic cells, and natural killer (NK) cells; LO was restricted to T lymphocytes. Gene-based analysis identified 46 EO vitiligo-associated, 56 LO vitiligo-associated, and 20 shared genes, revealing subtype-specific biological functions and tissue enrichment patterns. SMR and colocalization confirmed ribonuclease T2 (RNASET2) as an EO core target and granzyme B (GZMB)/thyrotroph embryonic factor (TEF) as LO targets. These targets demonstrated subtype-specific expression in peripheral blood single cells. Spatial mapping showed EO genes enriched in mesenchymal and extracellular matrix compartments, while LO genes localized to epidermal and keratinocyte regions. Exploratory validation confirmed RNASET2 downregulation in EO patients, while GZMB and TEF decreased in LO patients.

**Conclusions:**

This study reveals substantial genetic heterogeneity between age-stratified vitiligo subtypes. Through our multi-layered analytical approach, we identified subtype-specific risk genes, cellular mechanisms, and tissue microenvironmental contexts, thereby providing a molecular foundation for precision diagnostics and age-tailored therapeutic strategies in vitiligo management.

## Introduction

Vitiligo is a chronic acquired depigmentation disease, which is characterized by the selective destruction of epidermal melanocytes, leading to discrete, well-circumscribed, white macules and patches with a major effect on quality of life and psychosocial well-being (1, 2). The disease is found in 0.5-2% of the world population; it varies significantly by ethnicity and geographical area (3). Clinically, it is possible to divide vitiligo into early-onset (EO) and late-onset (LO), based on the age of onset (4). In epidemiological studies, there is always a bimodal onset distribution, with peaks in adolescence and middle adulthood that suggests possible etiologic heterogeneity of the two subtypes. Oxidative stress and melanocyte-intrinsic vulnerability play important roles in the pathogenesis of vitiligo and autoimmune mediated destruction (5, 6). Present evidences indicate that cluster of differentiation 8-positive (CD8+)cytotoxic T lymphocytes kills melanocytes by direct action of the interferon-gamma (IFN-γ) signaling axis, which has been proven as a therapeutic target by successfully treating using JAK inhibitors such as ruxolitinib (7–9). Nevertheless, there is still a lot to be learned regarding the particularities of vitiligo disease that lead to the observed phenotypic differences between the EO and LO types of this disease even though great strides have been made in deciphering the general course of vitiligo development. EO type vitiligo tends to manifest under the age of 20, has a greater extension of the skin involvement, and has greater familial aggregation, while LO vitiligo is commonly linked to organ specific autoimmunological disorders like thyroid disease and type I diabetes (10, 11). It remains to be determined whether these disparate clinical phenotypes represent fundamentally different modes of disease pathogenesis, and this question should be addressed systematically.

The genome-wide association studies (GWAS) have greatly improved our knowledge about genetic basis of vitiligo and most of these risk loci are located on immune-related and melanocyte-specific genes (12, 13). Recent advances in methodology have additionally facilitated more complex genetic analyses than traditional association testing.e.g., linkage disequilibrium score regression (LDSC), which enables estimating genetic correlations between traits and partitioning of heritability by functional annotation (14), while the multi-marker analysis of genomic annotation (MAGMA) enables gene based association analyses and pathway enrichment testing (15). Third, the integration of GWAS results with single cell and spatial transcriptome atlases can map the genetic risk to particular cell populations at an unprecedented resolution. These approaches have been successful at identifying the genetic architecture of subtypes within a heterogeneous disorder, e.g., for inflammatory bowel disease,rheumatoid arthritis, and migraine (16–18), which provided a good methodological basis for revealing the genetic mechanism of vitiligo.

To comprehensively characterize the genetic heterogeneity between EO and LO vitiligo, in this study, we performed systematic analyses as follows. We estimated genetic similarity among the subtypes via genetic correlations and examined functional annotation and cell-type-specific heritability enrichment by stratified LDSC. We used MAGMA, summary-data-based mendelian randomization (SMR) and colocalization analysis for identifying subtype-specific risk genes as well as possible therapeutic targets, and analyzed the expression specificity of core targets in peripheral blood immune cell types. We also incorporated spatial transcriptome information for mapping genetic risk variants into certain tissue niche. Overall, we demonstrate a high degree of genetic heterogeneity between the different forms of vitiligo as well as support the presence of key targets in their transcripts and proteins. Taken together, these results reveal possibly different genetic architectures for the age-stratified vitiligo phenotypes, as well as highlight putative therapeutic targets that could be pursued in specific subtypes, establishing a molecular basis of precision medicine approaches that account for the age-at-onset heterogeneity.

## Methods

**Figure 1:**
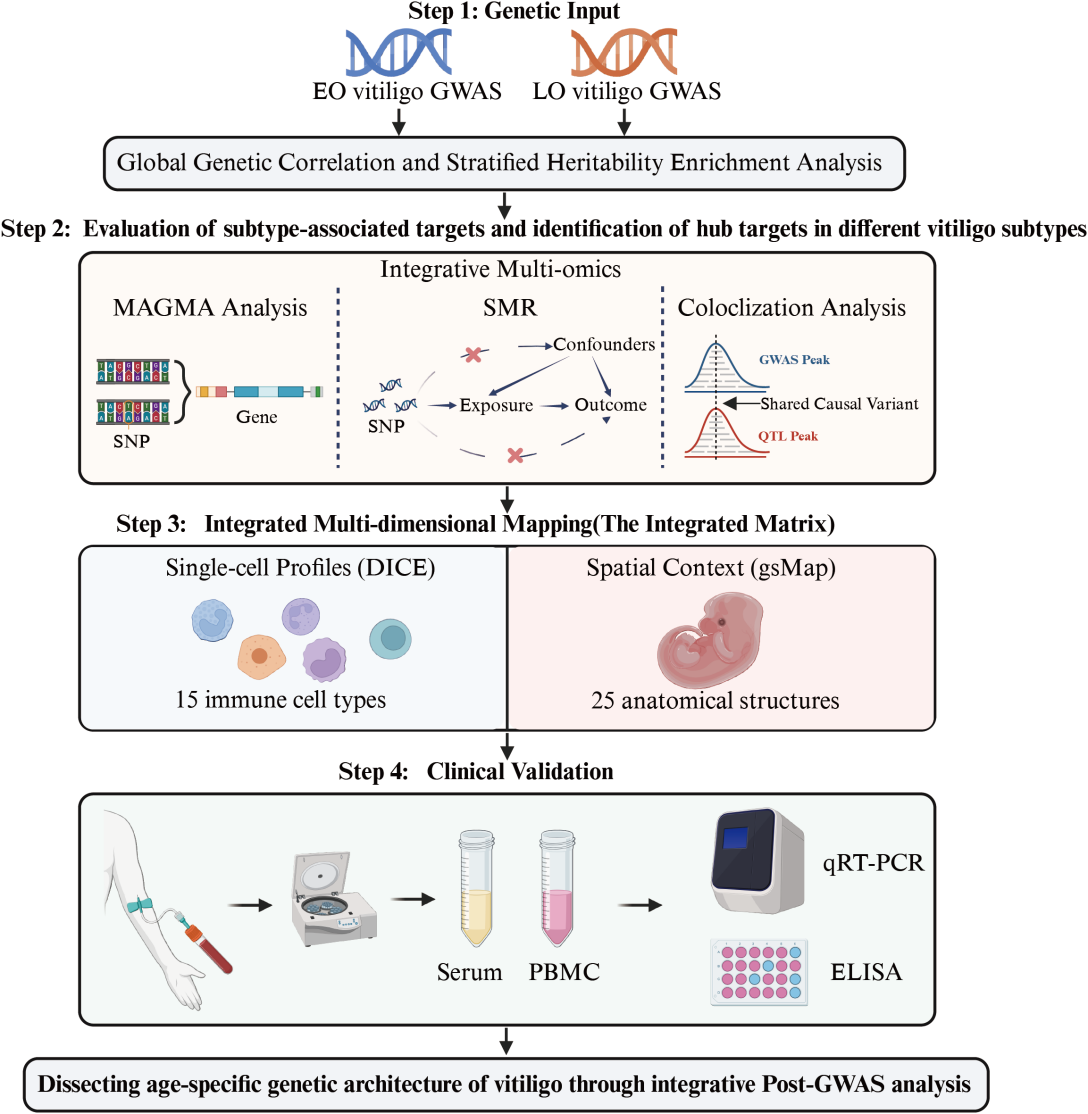
Workflow for dissecting age-specific genetic architecture of vitiligo through integrative post-GWAS analysis. EO, Early-Onset; LO, Late-Onset; GWAS, Genome-Wide Association Study; MAGMA, Multi-marker Analysis of GenoMic Annotation; SNP, Single Nucleotide Polymorphism; SMR, Summary-data-based Mendelian Randomization; QTL, Quantitative Trait Locus; DICE, Database of Immune Cell Expression; gsMap, gene-set Map; PBMC, Peripheral Blood Mononuclear Cells.

The GWAS summary statistics of EO and LO vitiligo were taken from Jin et al, which contained 4,523 vitiligo cases of European ancestry (19). After age-of-onset stratification, the EO vitiligo subgroup included 704 cases and 9,031 controls (mean age-of-onset: 10.3 years), while the LO vitiligo subgroup comprised 1,467 cases and 19,156 controls (mean age-of-onset: 34.0 years). The workflow for analysis is shown in **Figure 1**, **Supplementary Table 1** contains details about the data sources, while a summary description of the used software packages and analysis techniques can be found in **Supplementary Table 2**.

### Genetic correlation analysis

To estimate how much genetics is shared by EO and LO vitiligo, we estimated rg for these two traits with LDSC. LDSC uses LD scores derived from a reference dataset to compute an estimate of genetic correlation given GWAS summary statistics while decomposing correlation in terms of its genetic vs confounding component. We run analysis using LDSC both pre and post-fixing the LD score intercept, and we calculated statistical significance based on the *P-values* and standard error estimated by LDSC. To identify functional annotations and cell types enriched for heritability, we ran partitioned LDSC analyses on 53 baseline functional categories (20). Enrichment was defined as the ratio between the fraction of heritability explained by SNPs in a given category and the fraction of SNPs belonging to that category. We additionally tested cell type specific enrichments to chromatin accessibility data from the Corces Assay for Transposase-Accessible Chromatin using sequencing (ATAC-seq )data set, for a total of 13 different immune cell types (21, 22). We estimated the enrichment coefficients and *P-values* by LDSC. The statistical significance of all enrichments was evaluated with Bonferroni correction.

### Gene-based association and functional enrichment analysis

We performed gene-based association analysis using the MAGMA software (https://cncr.nl/research/magma/) which aggregates SNP-level associations into gene level statistics. MAGMA uses a multiple linear principle component regression model that accounts for linkage disequilibrium between SNPs in gene boundary. We only considered proteins coding genes, as we wanted to focus on more biologically interpretable and possibly druggable targets. Finally, the gene level *P-values* are adjusted for multiple testing controlling the family wise error rate.

To elucidate biological pathways of vitiligo susceptibility, we further carried out a functional enrichment analysis based on Kyoto Encyclopedia of Genes and Genomes (KEGG) pathway database for identifying over-represented biological process in significant-associated genes (23). We used MAGMA gene-tissue association results to determine tissue-level expression enrichment using RNAseq data available through the Genotype-Tissue Expression (GTEx) database (24), including 54 human tissues, to identify tissues in which the vitiligo associated genes show a preference of expression.

### Summary-data-based mendelian randomization analysis

SMR analysis was performed to evaluate putative associations between gene expression or protein abundance and vitiligo susceptibility (25). Significantly associated genes from MAGMA analysis were selected for SMR testing using GWAS summary statistics combined with expression quantitative trait loci (eQTL) data from the eQTLGen Consortium and protein quantitative trait loci (pQTL) data from the UK Biobank Pharma Proteomics Project (UKB-PPP) (26, 27). SMR uses top cis-QTL variants (defined as variants within ±1 MB of gene boundaries) as instrumental variables to determine if molecular trait-disease associations reflect shared variants. The heterogeneity in dependent instruments (HEIDI) test was applied to distinguish pleiotropy from linkage disequilibrium; HEIDI *P-values* > 0.01 suggests a pleiotropic effect rather than linkage confounding. Statistical significance thresholds were adjusted for the number of genes tested.

### Colocalization analysis

Bayesian colocalization analysis was performed for each prioritized gene-trait pair using the coloc package in R to evaluate whether GWAS and cis-QTL signals share a common variant. Colocalization evidence was quantified using posterior probabilities under two hypotheses: PPH3 representing both traits associated with distinct variants, and PPH4 representing both traits associated with a single shared variant. PPH3 + 4 > 0.8 indicated significant colocalization, with PPH4 > 0.8 providing strong evidence for a statistically shared variant driving both molecular and disease associations (28).

### Single-cell expression analysis

Cell-type-specific expression patterns of prioritized genes in peripheral blood were characterized using single-cell RNA-seq data from the Database of Immune Cell Expression (DICE) project (29). This dataset comprises 15 immune cell subsets including T cells, NK cells, B cells, and monocytes. Gene expression was quantified as transcripts per million (TPM) followed by log_2_ transformation, and cell-type-specific expression differences were assessed using Mann-Whitney U tests comparing each cell type against all others.

### Spatial transcriptome mapping

Disease-associated genes were mapped to specific tissue and organ contexts using the genetically informed spatial mapping of cells for complex traits (gsMap) framework, which integrates GWAS summary statistics with spatial transcriptome data from mouse embryonic development (E16.5) (30). This dataset encompasses 25 distinct anatomical structures and developmental stages, capturing spatially resolved gene expression patterns across developing tissues. The method calculates gene-spatial scores (GSS) that quantify the spatial expression specificity of disease-associated genes by integrating genetic association signals with spatial expression profiles. For each vitiligo subtype, enrichment Z-scores and *P-values* were computed for individual spatial spots, with tissue-level enrichment defined as *P-value* < 0.05. Gene-level spatial correlation was assessed by calculating Pearson correlation coefficients (PCC) between gene-disease association scores and spatial expression patterns across anatomical structures. Positive PCC values indicate genes preferentially expressed in disease-enriched tissues, whereas negative values reflect expression in non-disease tissues. Median GSS values were used to summarize spatial expression specificity for prioritized genes.

### Sample acquisition

Participants were categorized into two groups based on the age of onset: EO vitiligo (age of onset 6–14 years, n = 10) and LO vitiligo (age of onset 25–45 years, n = 10) (19). Simultaneously, age-matched healthy controls were recruited and divided into two groups: young controls (age-matched to EO vitiligo patients, n = 10) and adult controls (age-matched to LO vitiligo patients, n = 10). A total of 40 participants were included in this study.

Inclusion criteria for vitiligo patients were as follows: (1) clinical diagnosis of vitiligo confirmed by dermoscopy and/or Wood’s lamp examination; (2) age of onset 6–14 years for EO vitiligo or 25–45 years for LO vitiligo; (3) disease duration ≥ 6 months; and (4) no treatment with topical or systemic immunosuppressants, phototherapy, or corticosteroids within the past 4 weeks. Exclusion criteria for vitiligo patients included: (1) concomitant autoimmune diseases (e.g., autoimmune thyroiditis, rheumatoid arthritis, systemic lupus erythematosus); (2) acute or chronic infectious diseases; (3) malignant tumors; (4) pregnancy or lactation; and (5) severe hepatic, renal, or cardiovascular dysfunction. Inclusion criteria for healthy controls were: (1) no history of vitiligo or other dermatological disorders; (2) no history of autoimmune diseases; (3) no family history of vitiligo; and (4) no use of immunomodulatory drugs within the past 4 weeks. All participants provided written informed consent. The ethical approval for this study (Ethical Review No. L-28)) was granted by the Ethics Committee of the First Affiliated Hospital of Kunming Medical University on October 14, 2025. (Approval No.: (2025) Ethical Review No. L-287)and was conducted in accordance with the principles of the Declaration of Helsinki.

Fasting peripheral venous blood (5 mL) was collected from all participants into EDTA tubes for RNA extraction and serum separator tubes for ELISA. Serum was separated by centrifugation at 3000 rpm for 10 min at 4 °C within 2 hours of collection. All samples were aliquoted and stored at -80 °C until analysis.

### Quantitative reverse transcription PCR assay

Total RNA was extracted from peripheral blood mononuclear cells (PBMCs) using TRIzol reagent (Invitrogen, Carlsbad, CA, USA), and used to generate cDNA with the PrimeScript RT Reagent Kit (TaKaRa, RR037A). The cDNA was applied as template for qRT-PCR, which was carried out in an Applied Biosystems 7500 Real-Time PCR System (Thermo Fisher Scientifi) with TB Green Premix Ex Taq II (TaKaRa, RR820A) in a 40-cycle PCR. The following were gene-specific forward and reverse primer pairs: RNASET2: 5’-GAGTGATACCCAAAATCCAGT-3’ and 5’-GCTTAGTGAGGCACAGTTCT-3’, GZMB: 5’-TCAAAGAACAGGAGCCGACC-3’ and 5’-TTGGCCTTTCTCTCCAGCTG-3’, TEF: 5’-CAGTGTCTGCATGGGTAGCA-3’ and 5’-AGACAGCAGCCATCCTAGGA-3’, GAPDH: 5’-CCAGCAAGAGCACAAGAGGAAGAG-3’ and 5’-GGTCTACATGGCAACTGTGAGGAG-3’. The relative expression levels were calculated by the 2^−ΔΔCt^ method. The mRNA levels of each target gene were normalized to the levels of GAPDH.

### Enzyme-linked immunosorbent assay

ELISA assays Serum concentrations of RNASET2, GZMB, and TEF were determined using commercially available ELISA kits (KE00402, proteintech for RNASET2; DGZB00, R&D Systems for GZMB; and HT0124, Kendall Scientific for TEF) according to the manufacturers’ instructions. Briefly, serum samples and standards were added to pre-coated 96-well plates and incubated at 37 °C for 90 minutes. Following a washing step, biotinylated detection antibodies were added and incubated at 37 °C for 60 minutes, followed by the addition of HRP-conjugated Streptavidin for 30 minutes at 37 °C. After a final wash, the Tetramethylbenzidine(TMB) substrate solution was added and incubated in the dark for 15–20 minutes. The reaction was halted with a stop solution, and the optical density (OD) was measured at 450 nm using a microplate reader (Bio-Rad, USA). Protein concentrations were calculated based on the standard curves. All samples were analyzed in duplicate, and the mean values were used for analysis.

### Statistical analysis

Statistical analysis was performed using GraphPad Prism 10.6.1. Quantitative data are expressed as mean ± standard deviatio. The Shapiro-Wilk test was used to assess the normality of data distribution. For normally distributed data, comparisons among the four groups were performed using one-way analysis of variance (ANOVA) followed by Tukey’s *post-hoc* test for multiple comparisons. For non-normally distributed data, the Kruskal-Wallis test was employed, followed by Dunn’s *post-hoc* test. A two-tailed *P-value* < 0.05 was considered statistically significant.

## Results

### Genetic correlation and functional enrichment between vitiligo subtypes

LDSC analysis showed significant genetic correlation between EO and LO vitiligo (constrained: rg = 0.672, *P-value* = 7.418 × 10^-7^; unconstrained: rg = 0.787, *P-value* = 1.334 × 10^-41^). While substantial shared genetic architecture exists, the genetic correlation deviated significantly from unity (rg < 1), indicating meaningful genetic heterogeneity between subtypes (**Supplementary Table 3**).

**Figure 2:**
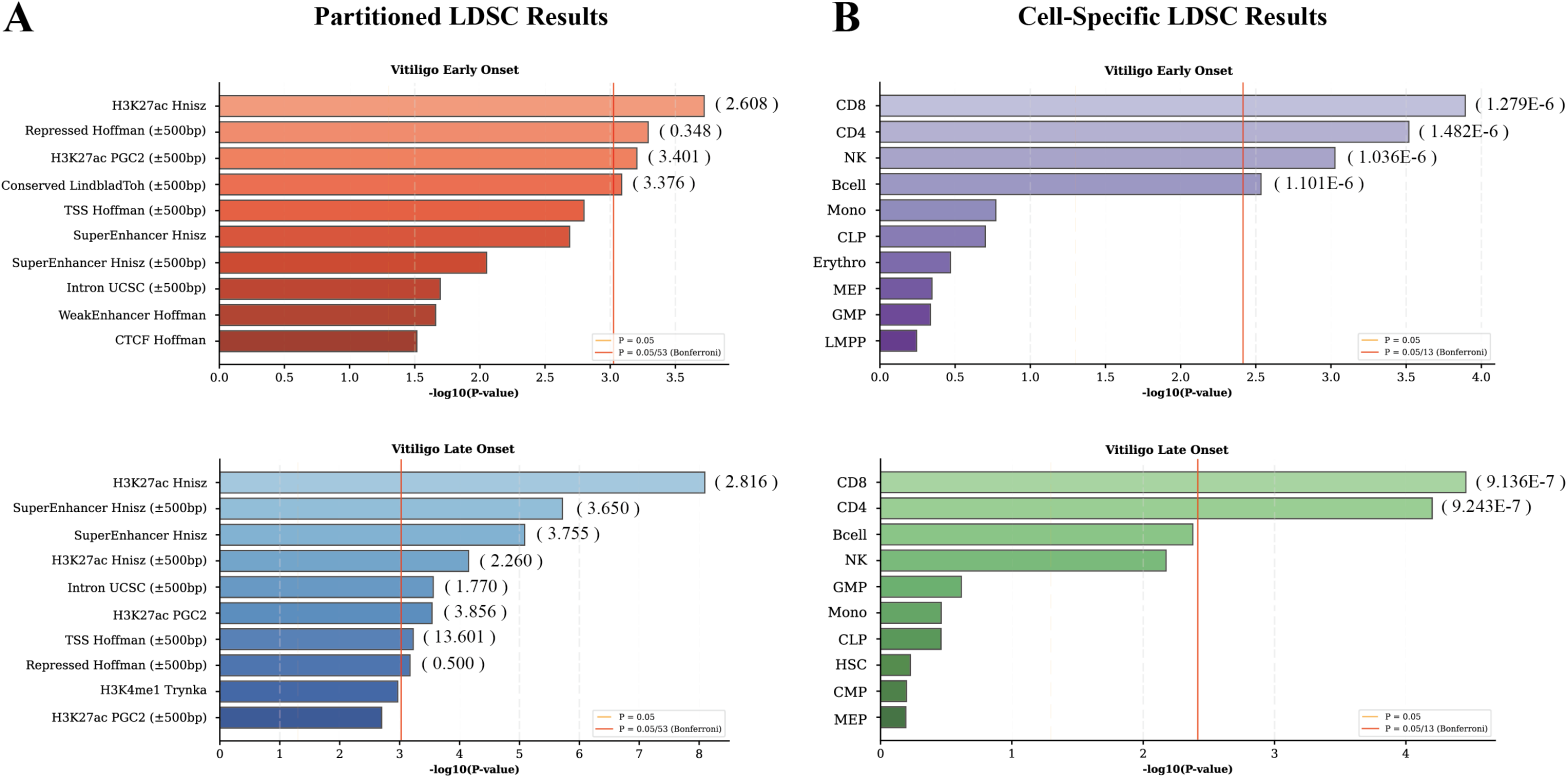
Functional annotation and cell-type-specific heritability enrichment analysis. **(A)** Partitioned linkage disequilibrium score regression (LDSC) results showing functional annotation enrichment in early-onset (top panel) and late-onset (bottom panel) vitiligo. Enrichment scores for each annotation are indicated in parentheses. **(B)** Cell-type-specific heritability enrichment analysis using chromatin accessibility data from 13 immune cell types in early-onset (top) and late-onset (bottom) vitiligo. The coefficients for each cell type are shown in parentheses.

Partitioned LDSC identified overlapping yet distinct functional enrichment patterns (significance threshold was 0.05/53 = 9.434 × 10^-4^). Both subtypes exhibited significant enrichment in H3K27ac-marked regions, a hallmark of active enhancers. EO vitiligo showed enrichment in conserved regions (*P-value* = 8.158 × 10^-4^), H3K27ac-marked regions (*P-value* = 1.899 × 10^-4^), and H3K27ac PGC2 marks (*P-value* = 6.240 × 10^-4^), with depletion in repressed chromatin (*P-value* = 5.115 × 10^-4^). LO vitiligo displayed broader regulatory enrichment: H3K27ac regions (*P-value* = 8.042 × 10^-9^; *P-value* for extend = 7.053 × 10^-5^; *P-value* for PGC2 marks = 2.886 × 10^-4^), super-enhancers (*P-value* = 8.165 × 10^-6^; *P-value* for extend = 1.910 × 10^-6^), transcription start sites (*P-value* = 5.914 × 10^-4^), and intronic regions (*P-value* = 2.740 × 10^-4^), alongside depletion in repressed regions (*P-value* = 6.703 × 10^-4^). This pattern suggests more complex transcriptional dysregulation in late-onset disease (**Figure 2A**, **Supplementary Table 4**). Cell-type-specific analysis revealed immune cell enrichment in both subtypes (significance threshold was 0.05/13 = 3.846 × 10^-3^). EO vitiligo exhibited enrichment in CD8+ T cells (*P-value* = 1.278 × 10^-4^), CD4+ T cells (*P-value* = 3.033 × 10^-4^), NK cells (*P-value* = 9.410 × 10^-4^), and B cells (*P-value* = 2.921 × 10^-3^). LO vitiligo showed enrichment specifically in CD8+ (*P-value* = 3.487 × 10^-5^) and CD4+ T cells (*P-value* = 6.297 × 10^-5^), with more pronounced T cell-centric patterns highlighting the central role of adaptive immunity (**Figure 2B**, **Supplementary Table 5**).

Collectively, EO and LO vitiligo exhibited distinct heritability enrichment patterns. EO vitiligo showed enrichment in conserved regulatory elements with broader immune cell involvement, while LO vitiligo displayed more extensive regulatory enrichment across super-enhancers and transcription start sites with concentrated T cell-specific signals.

### Gene-level associations and enrichment analysis between vitiligo subtypes

**Figure 3:**
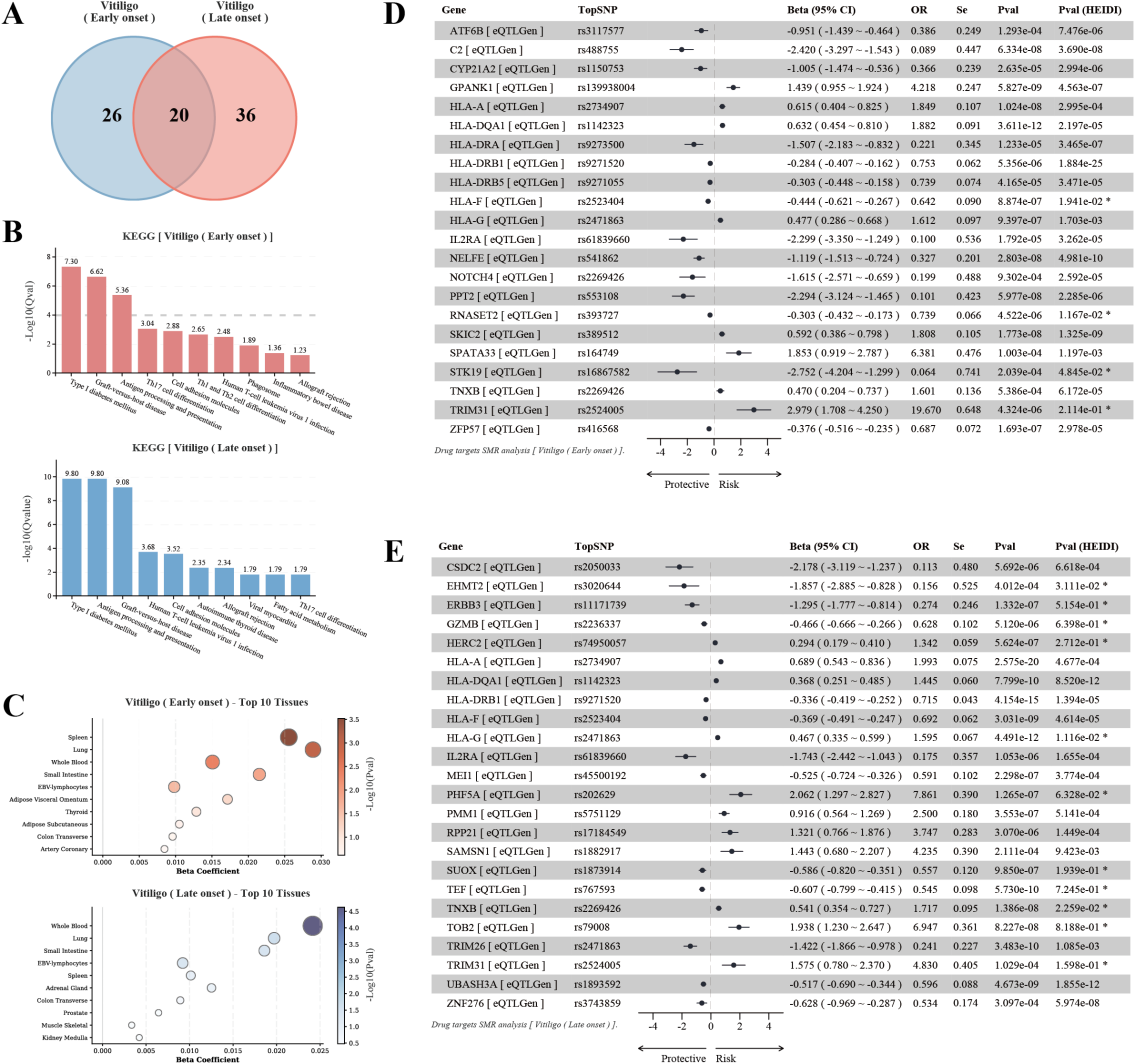
Identification and functional characterization of vitiligo subtype-associated genes. **(A)** Venn diagram showing overlap of genome-wide significant genes between EO (26 unique genes), LO (36 unique genes), and shared genes (20 genes) identified by MAGMA analysis. **(B)** KEGG pathway enrichment analysis for EO vitiligo (top panel) and LO vitiligo (bottom panel). The y-axis shows enriched pathways and the x-axis represents -log10Q. **(C)** Tissue-specific expression enrichment of vitiligo-associated genes in GTEx tissues. Top 10 tissues for EO vitiligo (top panel) and LO vitiligo (bottom panel) are shown. **(D)** Summary-data-based Mendelian randomization (SMR) analysis results for EO vitiligo at the transcriptional level using eQTL data from eQTLGen Consortium. Forest plots show beta coefficients with 95% confidence intervals, odds ratios (OR), standard errors (Se), *P-values*, and HEIDI test *P-values*. Asterisks (*) indicate HEIDI *P-value* > 0.01, suggesting shared variants rather than linkage disequilibrium. **(E)** SMR analysis results for LO vitiligo at the transcriptional level. Format is identical to **(D)**.

MAGMA gene-based association analysis identified 46 genome-wide significant genes for EO vitiligo (significance threshold was 0.05/17414 = 2.871 × 10^-6^) and 56 for LO vitiligo (significance threshold was 0.05/16590 = 3.014 × 10^-6^), with 20 genes shared between subtypes (**Figure 3A**). Overlapping genes included major histocompatibility complex components (HLA-A, HLA-F, HLA-G, HLA-DQA1, HLA-DRA, HLA-DRB1, HLA-DRB5), immune regulatory genes (IL2RA), epigenetic modifiers (EHMT2, ATF6B), and complement pathway components (TNXB, CYP21A2), reflecting both shared and distinct genetic underpinnings (**Supplementary Tables 6**, **7**).

Functional and tissue-specific enrichment analyses revealed substantial overlap alongside notable distinctions between subtypes. KEGG pathway enrichment identified shared autoimmune mechanisms including Type I diabetes mellitus, graft-versus-host disease, antigen processing and presentation, T cell differentiation pathways, and cell adhesion molecules. However, LO vitiligo uniquely exhibited fatty acid metabolism enrichment, with 17 significant pathways compared to 9 in EO vitiligo, suggesting broader functional dysregulation in late-onset disease (**Figure 3B**; **Supplementary Tables 8**, **9**). Tissue-specific enrichment analysis showed immune system involvement in both subtypes (significance threshold was 0.05/54 = 9.260 × 10^-4^), with spleen reaching statistical significance in EO vitiligo (*P-value* = 3.047 × 10^-4^) and whole blood in LO vitiligo (*P-value* = 2.366 × 10^-5^), while other immune tissues showed nominal enrichment (**Figure 3C**; **Supplementary Tables 10**, **11**). These results parallel the genetic correlation patterns observed in LDSC analysis, revealing both shared immunopathogenic foundations and distinct functional signatures between subtypes.

### Identification of potential core targets

To evaluate whether core therapeutic targets differ between vitiligo subtypes, we performed SMR analysis integrating eQTLGen data with GWAS summary statistics. This analysis identified four genes significantly associated with EO vitiligo (HLA-F, TRIM31, RNASET2, STK19) and eleven genes with LO vitiligo (HLA-G, TEF, TNXB, TOB2, PHF5A, ERBB3, HERC2, SUOX, GZMB, TRIM31, EHMT2). All genes passed significance thresholds for both SMR testing (significance threshold was 0.05/46 = 1.087 × 10–^3^ for EO vitiligo; significance threshold was 0.05/56 = 8.929 × 10–^4^ for LO vitiligo) and HEIDI filtering, suggesting potential associations rather than linkage confounding (**Figures 3D-E**; **Supplementary Tables 12**, **13**). To assess translational-level robustness, we performed proteome-wide SMR using pQTL data from UKB-PPP, which identified HLA-A, AGER, EGFL7 and RNASET2 for EO vitiligo, and C1QTNF6, CCDC134, GZMB, TEF, SUOX, and IL2RA for LO vitiligo (**Table 1**; **Supplementary Tables 14**, **15**). RNASET2 replicated across transcriptome and proteome in EO vitiligo, while GZMB, TEF, and SUOX showed cross-omic replication in LO vitiligo. However, SUOX exhibited opposite effect directions at transcriptome versus proteome levels, indicating complex regulation that precludes clear interpretation. We therefore prioritized RNASET2 for EO vitiligo and GZMB and TEF for LO vitiligo as high-confidence statistically prioritized candidates. RNASET2 showed protective effects in EO vitiligo at transcriptome (OR = 0.739, Se = 0.066, *P-value* = 4.522 × 10^-6^) and proteome (OR = 0.528, Se = 0.163, *P-value* = 8.652 × 10^-5^) levels. In LO vitiligo, GZMB displayed protective associations at transcriptome (OR = 0.628, SE = 0.102, *P-value* = 5.120 × 10^-6^) and proteome (OR = 0.522, Se = 0.127, *P-value* = 3.110 × 10^-7^) levels, while TEF showed consistent effects across transcriptome (OR = 0.545, Se = 0.098, *P-value* = 5.730 × 10^-10^) and proteome (OR = 0.024, Se = 0.755, *P-value* = 7.745 × 10^-7^) levels. .

**Table 1:** Protein-level Mendelian randomization analysis for vitiligo subtypes.

Gene	TopSNP	Beta (95% CI)	OR	Se	Pval	Pval (HEIDI)
Vitiligo (Early onset)						
AGER [UKBPPP]	rs34562262	-1.390 (-2.304 ~ -0.476)	0.249	0.466	2.886E-03	0.100*
C2 [UKBPPP]	rs115032094	0.836 (0.215 ~ 1.458)	2.307	0.317	8.378E-03	0.002
CFB [UKBPPP]	rs641153	0.790 (0.514 ~ 1.066)	2.203	0.141	2.062E-08	0.000
CHMP1A [UKBPPP]	rs460879	-2.270 (-3.514 ~ -1.027)	0.103	0.635	3.469E-04	0.001
DPEP1 [UKBPPP]	rs409170	0.142 (-0.005 ~ 0.289)	1.153	0.075	5.882E-02	0.000
EGFL7 [UKBPPP]	rs74557797	-0.063 (-0.759 ~ 0.634)	0.939	0.355	8.603E-01	0.030*
HLA-A [UKBPPP]	rs1632908	-0.476 (-0.648 ~ -0.304)	0.621	0.088	5.634E-08	0.051*
HLA-DRA [UKBPPP]	rs36096565	0.268 (0.059 ~ 0.476)	1.307	0.107	1.198E-02	0.002
IL2RA [UKBPPP]	rs12722497	-0.225 (-0.427 ~ -0.024)	0.798	0.103	2.851E-02	0.004
RNASET2 [UKBPPP]	rs3756838	-0.640 (-0.959 ~ -0.320)	0.528	0.163	8.652E-05	0.046*
TNXB [UKBPPP]	rs114989909	-0.552 (-1.052 ~ -0.052)	0.576	0.255	3.034E-02	0.000
Vitiligo (Late onset)						
C1QTNF6 [UKBPPP]	rs5750394	-2.822 (-4.251 ~ -1.393)	0.059	0.729	1.083E-04	0.179*
CCDC134 [UKBPPP]	rs28384790	-1.512 (-2.941 ~ -0.082)	0.221	0.729	3.821E-02	0.010*
CHMP1A [UKBPPP]	rs460879	-1.294 (-2.143 ~ -0.446)	0.274	0.433	2.778E-03	0.003
ERBB3 [UKBPPP]	rs2292238	-1.253 (-1.913 ~ -0.593)	0.286	0.337	1.994E-04	0.000
GZMB [UKBPPP]	rs8192917	-0.651 (-0.900 ~ -0.401)	0.522	0.127	3.110E-07	0.072*
HLA-A [UKBPPP]	rs1632908	-0.219 (-0.330 ~ -0.109)	0.803	0.056	1.031E-04	0.005
HLA-DRA [UKBPPP]	rs36096565	0.429 (0.288 ~ 0.569)	1.535	0.072	2.493E-09	0.001
IL2RA [UKBPPP]	rs12722497	-0.214 (-0.354 ~ -0.073)	0.808	0.072	2.876E-03	0.040*
SUOX [UKBPPP]	rs773125	4.005 (2.178 ~ 5.832)	54.868	0.932	1.730E-05	0.301*
TEF [UKBPPP]	rs4822027	-3.731 (-5.211 ~ -2.251)	0.024	0.755	7.745E-07	0.266*
TG [UKBPPP]	rs75143612	-1.052 (-2.272 ~ 0.169)	0.349	0.623	9.134E-02	0.009
TNXB [UKBPPP]	rs114989909	-0.538 (-0.848 ~ -0.229)	0.584	0.158	6.428E-04	0.000
TRIM40 [UKBPPP]	rs17184549	-1.233 (-1.704 ~ -0.762)	0.291	0.240	2.878E-07	0.001

The table shows genes with significant associations for EO vitiligo and LO vitiligo. Columns include: Gene name with data source, top instrumental SNP (TopSNP), effect size with 95% confidence interval (Beta (95% CI)), odds ratio (OR), standard error (Se), SMR *P-value*, and HEIDI test *P-value*. Asterisks (*) indicate HEIDI *P-value* > 0.01, suggesting the association is driven by a shared variant rather than linkage disequilibrium. Negative beta values indicate protective effects, while positive values indicate risk-increasing effects.

**Figure 4:**
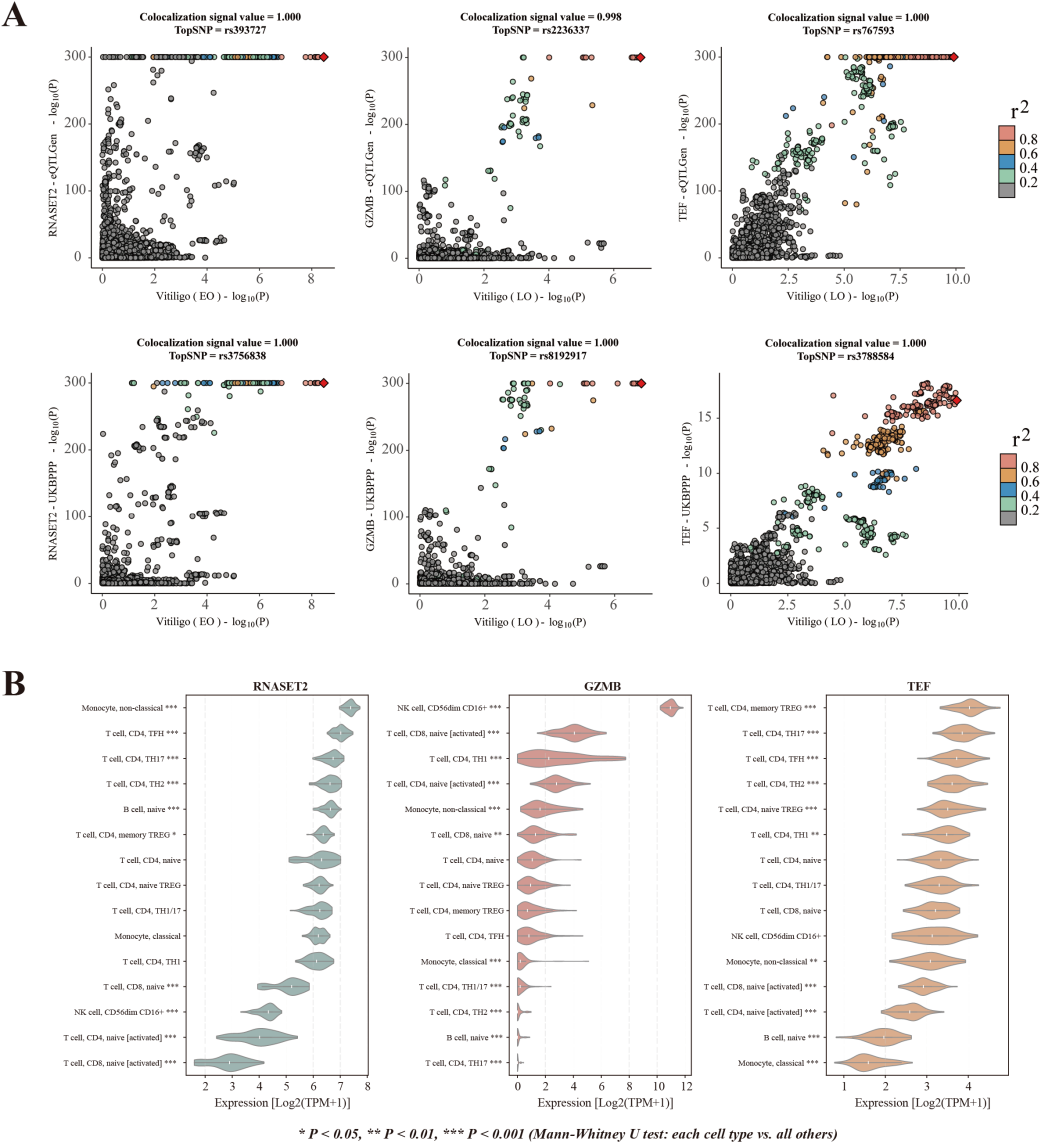
Colocalization analysis and cell-type-specific expression of core target genes. **(A)** Bayesian colocalization scatter plots for prioritized gene-trait pairs. Each plot shows the association between vitiligo GWAS signals (x-axis, -log10P) and gene expression/protein abundance (y-axis, -log10P). Top row: RNASET2 with EO vitiligo (eQTL and pQTL); GZMB with LO vitiligo (eQTL); TEF with LO vitiligo (eQTL). Bottom row: RNASET2 with EO vitiligo (pQTL); GZMB with LO vitiligo (pQTL); TEF with LO vitiligo (pQTL). Points are colored by linkage disequilibrium (r²) with the top SNP. Colocalization signal values and top SNPs are indicated above each plot. **(B)** Cell-type-specific expression patterns of RNASET2 (left), GZMB (middle), and TEF (right) across 15 immune cell subsets from peripheral blood, derived from the DICE (Database of Immune Cell Expression, eQTLs, and Epigenomics) database. This dataset comprises transcriptomic data generated from 13 immune cell types and 2 activated cell types isolated from 106 leukapheresis samples provided by 91 healthy donors, encompassing 12,254 eGenes identified across these cell populations. Violin plots show expression levels as log2(TPM + 1). Cell types are ordered by median expression level. Statistical significance was assessed using Mann-Whitney U test comparing each cell type versus all others (*P-value < 0.05, **P-value < 0.01, ***P-value < 0.001).

Colocalization analysis supported the associations between the three prioritized genes and the two vitiligo subtypes through shared genetic signals. For EO vitiligo, RNASET2 showed significant colocalization at both transcriptome (PPH3 + 4 = 1.000) and proteome (PPH3 + 4 = 1.000) levels. For LO vitiligo, GZMB exhibited strong colocalization at both transcriptome (PPH4 = 0.843) and proteome (PPH4 = 0.991) levels, while TEF demonstrated similar patterns (eQTL: PPH4 = 0.942; pQTL: PPH4 = 0.891). Notably, heterogeneity in top SNPs was observed across eQTL and pQTL colocalization analyses for the same gene-trait combinations, suggesting that gene-disease associations explained by shared variants may exhibit differences between transcriptional and translational levels. Nevertheless, these findings strengthened the genetic evidence provided by gene regulatory traits (**Figure 4A**, **Supplementary Table 16**).

### Cell-type-specific expression profiles of core therapeutic targets

Single-cell RNA-seq analysis from the DICE database revealed distinct expression patterns for the prioritized genes across 15 immune cell types, largely consistent with cell-type enrichment patterns identified in partitioned heritability analysis (**Figure 4B**). RNASET2, the high-confidence target for EO vitiligo, displayed highest expression in non-classical monocytes, T follicular helper cells, Th17 cells, Th2 cells, and naive B cells (all *P-values* < 0.001), with lower expression in CD8+ T cells and NK cells. This broad expression pattern across antigen-presenting cells and helper T cell subsets aligns with EO vitiligo’s enrichment in multiple immune cell types and supports RNASET2’s role in immune regulation and RNA metabolism. GZMB, prioritized for LO vitiligo, exhibited pronounced cell-type specificity with predominant expression in NK cells (*P-value* < 0.001), followed by activated CD8+ T cells and Th1 cells, while showing minimal expression in Th2, Th17, and B cells. TEF, the second LO target, was predominantly expressed in CD4+ T cell subsets, particularly memory regulatory T cells, Th17 cells, T follicular helper cells, Th2 cells, naive regulatory T cells, and Th1 cells (all *P-values* < 0.01), with reduced expression in activated T cells, B cells, and classical monocytes. The concentrated expression of both GZMB and TEF in T cell compartments mirrors LO vitiligo’s T cell-centric heritability enrichment, reinforcing the central role of adaptive immunity in late-onset disease pathogenesis.

### Spatial transcriptome mapping reveals tissue-specific pathogenic contexts

**Figure 5:**
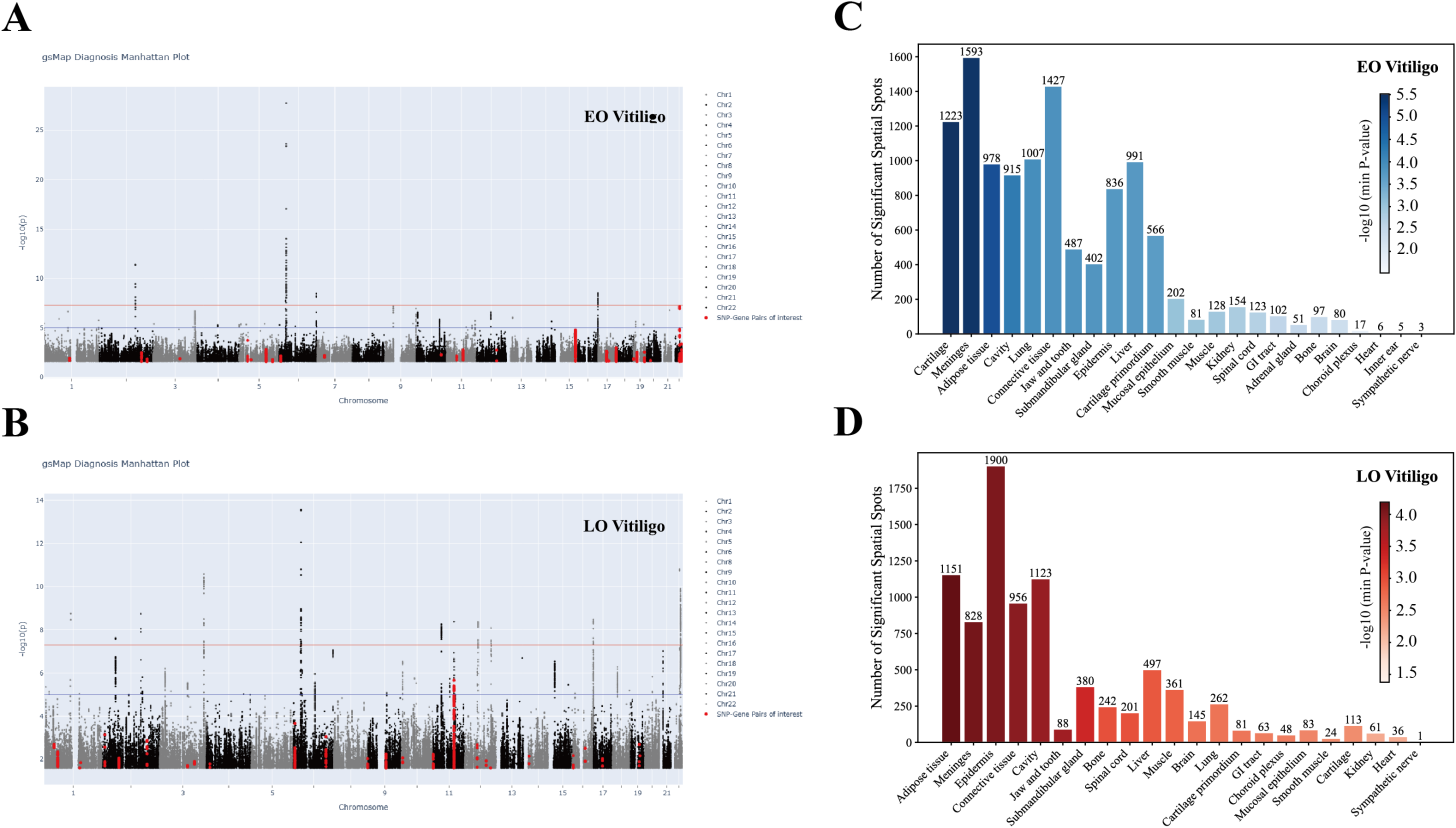
Spatial transcriptomic mapping of vitiligo risk genes. **(A)** Genome-wide spatial association signals of EO vitiligo-associated genes identified by genetically informed spatial mapping of cells for complex traits (gsMap). Manhattan plot shows chromosomal positions (x-axis) and -log10P (y-axis). Red points indicate SNP-gene pairs of interest showing significant spatial enrichment. Horizontal line represents genome-wide significance threshold. **(B)** Genome-wide spatial association signals of LO vitiligo-associated genes identified by gsMap. Format is identical to panel A. **(C)** Spatial spot counts colored by minimal P-value for EO vitiligo across 24 tissues. Bar height indicates the number of significant spatial spots (enrichment P-value < 0.05) per tissue, with exact counts labeled above each bar. Bar color represents the -log10(minimum P-value) for each tissue, as shown in the color scale. **(D)** Spatial spot counts colored by minimal P-value for LO vitiligo across 22 tissues. Format is identical to panel C.

**Figures 5A, B** illustrates the genome-wide spatial association signals of EO vitiligo and LO vitiligo related genes identified by the gsMap algorithm. Spatial transcriptome analysis using gsMap revealed distinct tissue enrichment patterns between vitiligo subtypes. EO vitiligo demonstrated enrichment in 11,474 spatial spots across 24 tissues, with strongest signals in cartilage (1,223 spots, Pmin-value = 3.045 × 10-6), meninges (1,593 spots, Pmin-value = 3.396 × 10-6), and adipose tissue (978 spots, Pmin-value = 1.014 × 10-5) (**Figure 5C**). LO vitiligo showed enrichment in 8,645 spatial spots, with prominent signals in adipose tissue (1,151 spots, Pmin-value = 6.444 × 10-5), meninges (828 spots, Pmin-value = 8.434 × 10-5), and epidermis (1,900 spots, Pmin-value = 9.216 × 10-5) (**Figure 5D**). The markedly higher epidermal involvement in LO vitiligo suggests that EO disease predominantly involves mesenchymal tissues while LO disease targets epidermal compartments, indicating that early-onset pathogenesis may originate in developmentally earlier tissue contexts whereas late-onset disease affects more differentiated epithelial structures.

Gene-level spatial mapping revealed contrasting molecular signatures. EO vitiligo displayed enrichment of extracellular matrix and structural proteins including SPARC (adipose tissue, PCC = 0.369), PPIC (cartilage primordium, PCC = 0.359), SERPINH1 (cartilage primordium, PCC = 0.355), and collagen genes COL1A2 and COL1A1 (adipose tissue, PCC = 0.352), reflecting mesenchymal remodeling processes. LO vitiligo exhibited keratinocyte-related gene enrichment with KRTDAP (epidermis, PCC = 0.355), KRT10 (epidermis, PCC = 0.339), DMKN (epidermis, PCC = 0.331), and PERP (epidermis, PCC = 0.327), indicating epidermal barrier dysfunction as a central feature of late-onset disease (**Supplementary Table 17**).

Spatial localization of therapeutic targets complemented tissue-specific enrichment patterns observed in MAGMA analysis (**Supplementary Table 18**). RNASET2 localized to adrenal gland (median GSS = 2.324), paralleling EO vitiligo’s significant spleen enrichment and consistent with immune regulatory mechanisms operating in primary lymphoid and endocrine tissues during early development. TEF showed expression in sympathetic nerve (median GSS = 1.869), suggesting involvement of more complex neuroimmune regulatory mechanisms characteristic of mature, systemically distributed immune responses. These spatial patterns indicate that EO vitiligo may engage immune mechanisms rooted in earlier developmental contexts, while LO vitiligo reflects pathogenic processes involving fully differentiated tissues and more sophisticated regulatory networks.

### Exploratory validation of biomarkers

**Figure 6:**
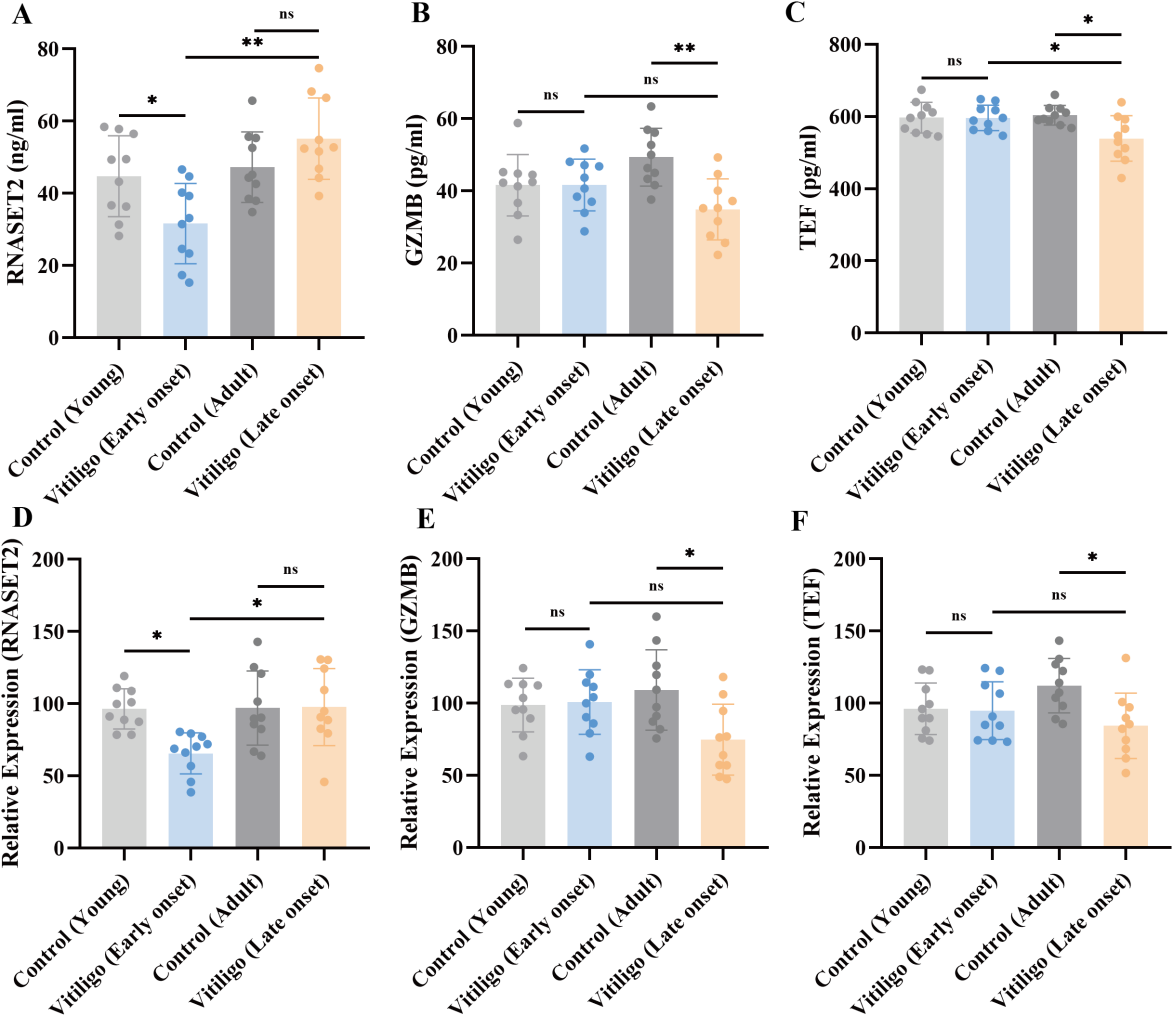
Expression levels of RNASET2, TEF, and GZMB in blood samples from patients with early-onset and late-onset vitiligo. **(A-C)** Serum concentrations of RNASET2 **(A)**, GZMB **(B)**, and TEF **(C)** were measured by ELISA. **(D-F)** Relative mRNA expression levels of RNASET2 **(D)**, GZMB **(E)**, and TEF **(F)** in PBMCs were determined by qRT-PCR. Data are presented as mean ± SD, n = 10. *P-value < 0.05, **P-value < 0.01, ns indicates no significant difference.

To validate the subtype-specific biomarkers identified through SMR analysis, we collected peripheral blood samples from patients with EO vitiligo, LO vitiligo. The expression levels of RNASET2, GZMB, and TEF were assessed by qRT-PCR and ELISA (**Figure 6**). RNASET2, the high-confidence target for EO vitiligo, showed significantly decreased mRNA expression in EO vitiligo patients compared to young controls (*P-value* < 0.05), with no significant difference observed between LO vitiligo patients and adult controls (**Figure 6A**). Consistently, serum RNASET2 protein levels were significantly reduced in EO vitiligo patients (*P-value* < 0.05), while LO vitiligo patients exhibited comparable levels to controls (**Figure 6D**). For the LO vitiligo targets, GZMB demonstrated significantly decreased mRNA and protein expression specifically in LO vitiligo patients compared to adult controls (*P-value* < 0.05 for qRT-PCR; *P-value* < 0.01 for ELISA), with no significant alterations in EO vitiligo patients (**Figures 6B, E**). Similarly, TEF showed significantly reduced mRNA and serum protein levels exclusively in LO vitiligo patients (*P-value* < 0.05), while no significant changes were detected in EO vitiligo patients (**Figures 6C, F**). These results provide exploratory clinical validation for the subtype-specific molecular signatures identified through multi-omics integration.

## Discussion

The partial genetic overlap between EO and LO vitiligo with rg ranging from 0.672 to 0.787 is reminiscent of the genetic architecture documented between Crohn’s disease and ulcerative colitis where shared immune pathways coexist with subtype specific disease mechanisms (16). Both subtypes draw from a common pool of immune susceptibility variants while retaining distinct genetic architectures that modulate phenotypic expression and disease course. The divergent heritability enrichment patterns carry important implications beyond simple annotation. EO vitiligo heritability is concentrated in evolutionarily conserved sequences which is consistent with the greater familial aggregation and earlier penetrant disease observed clinically (31). This suggests that EO susceptibility may be encoded more rigidly in the genome and is less susceptible to environmental modification. Conversely LO vitiligo heritability is distributed broadly across super enhancers, transcription start sites, active enhancers marked by H3K27ac, and DNase hypersensitivity sites. Super enhancers are large active chromatin structures that integrate transcription factor networks and orchestrate cell type specific transcriptional programs. They exhibit stimulus responsive plasticity and can be reprogrammed by environmental exposures, aging associated epigenetic drift, and inflammatory signals (32). The concentration of LO vitiligo heritability in these regulatory elements suggests that super enhancer dysregulation may be a defining mechanistic feature of late onset disease. This potentially explains why cumulative environmental exposures, age related changes in chromatin accessibility, and comorbid autoimmune conditions contribute disproportionately to LO vitiligo. Recent epigenomic studies in vitiligo have documented aberrant DNA methylation, dysregulated histone modifications, and non coding RNA disturbances as active contributors to disease pathogenesis (33, 34). These genetic findings lay the groundwork for future research indicating that integrated single cell transcriptomic and epigenomic profiling could better elucidate the divergent etiologies between early and late onset vitiligo populations.

The cell type specific heritability analysis further distinguishes these subtypes by revealing that EO vitiligo engages a broader spectrum of immune cell populations including dendritic cells, monocytes, NK cells, CD8 positive T cells, CD4 positive T cells, and B cells (35–37). In contrast LO vitiligo shows concentrated enrichment restricted primarily to CD8 and CD4 positive T lymphocytes. This distinction suggests that early onset pathogenesis involves a more generalized immune activation spanning both innate proximal and adaptive compartments whereas late onset disease is driven by more focused adaptive immune dysfunction. Innate immune cells including dendritic cells and NK cells are key initiators of vitiligo pathogenesis through melanocyte antigen presentation and interferon gamma production which link environmental triggers to adaptive immunity (38). Their greater genetic involvement in EO disease implies that upstream innate immune dysregulation represents an earlier and more prominent disease initiating event in children and adolescents. The overall trend implies that innate immune dysfunction may play a more pivotal role in EO vitiligo while adaptive immunity remains dominant in LO vitiligo. These distinct immune architectures highlight varying pathomechanisms between the two clinical subtypes and carry important treatment related implications for developing targeted therapeutic strategies.

The MHC locus located in 6p21 is home to the most significant genetic risk factors for vitiligo, including class I loci HLA-A and class II loci HLA-DRB1, HLA-DQB1) loci consistently implicated across populations (39, 40). We found common HLA association in subtypes, with shared genes including HLA-A, HLA-F, HLA-G, HLA-DQA1, HLA-DRA, HLA-DRB1, and HLA-DRB5 identified by MAGMA analysis, confirming the key role for antigen presentation in vitiligo pathology (41, 42). Interestingly, it was shown previously that certain HLA-DRB1-DQB1 haplotypes are associated with an earlier age of onset and the carrier develops vitiligo about 13 years sooner than a noncarrier (43). The variants were fine mapped into transcriptional enhancers which upregulated the HLA expression, suggesting a gain-of-function mechanism whereby increased antigen presentation leads to autoreactive T cell activation (44). Our detection of common as well as subtype-enriched HLA signal is consistent with a model whereby common HLA risk variants set the stage for disease susceptibility, while other, more subtype-specific variants modulate age of onset and clinical phenotype.

Among the most compelling candidates for an EO vitiligo-specific gene and potential therapeutic target is RNASET2, located at the 6q27 locus, which was previously implicated in EO vitiligo GWAS (39). RNASET2 encodes an evolutionarily conserved T2 ribonuclease that participates in both innate immune and cellular stress responses (45). Previous *in vitro* studies demonstrated that acute, stress-induced overexpression of RNASET2 in primary melanocytes and keratinocytes promotes apoptosis through physical interaction with TRAF2 and activation of caspase-dependent pathways, suggesting that elevated RNASET2 activity may play a pathogenic role in vitiligo (46). However, our SMR analysis reveals that genetic variants associated with higher RNASET2 expression exert a protective effect against EO vitiligo (OR = 0.739 at the transcriptome level, OR = 0.528 at the proteome level), and our clinical validation demonstrates that both RNASET2 mRNA in peripheral blood and serum RNASET2 protein levels are significantly decreased in EO vitiligo patients compared to age-matched healthy controls, whereas no significant differences are observed in LO vitiligo patients. These findings are not contradictory; rather, they reflect the context-dependent and tissue-compartment-specific duality of RNASET2 biology. Beyond its cell-autonomous pro-apoptotic role under acute oxidative stress, RNASET2 serves as a critical regulator of systemic innate immune homeostasis; silencing RNASET2 in macrophages downregulates M1 markers while upregulating M2 markers, thereby impairing immune surveillance capacity (47, 48). In neutrophils, RNASET2 co-localizes with primary granules and is incorporated into neutrophil extracellular traps during inflammatory stimulation (49). Therefore, the reduction in circulating RNASET2 levels observed in EO patients most plausibly reflects an impairment of systemic innate immune homeostasis. We propose that in genetically susceptible individuals with constitutively low RNASET2-mediated innate immune regulation, dendritic cells and monocytes fail to adequately resolve innate immune responses following melanocyte stress signaling, establishing a chronically permissive inflammatory environment that facilitates the early priming of autoreactive T cells from childhood. Locally, when melanocytes encounter oxidative stressors, acute stress-induced RNASET2 upregulation may occur transiently within the skin microenvironment as a cell-autonomous apoptotic signal (46); importantly, this local event is unlikely to be captured within the circulating compartment measured by our assays. Ultimately, this dual-compartment model reconciles the apparent contradiction and highlights the restoration of systemic RNASET2 levels as a mechanistically plausible therapeutic strategy, indicating that future clinical interventions may require precision targeting tailored to distinct tissue compartments.

The identification of GZMB as an LO-specific putative core target is mechanistically important but requires cautious reinterpretation in light of our finding of reduced GZMB levels in LO vitiligo patients. While granzyme B is classically known for its role in cytotoxic lymphocyte-mediated apoptosis, growing evidence highlights its essential function within regulatory T cells (Tregs) as a contact-dependent suppressive effector critical for maintaining immune homeostasis and controlling autoreactive T cell responses (50, 51). Critically, Giri et al. demonstrated that decreased GZMB transcripts are directly associated with reduced Treg suppressive capacity in generalized vitiligo patients, providing direct molecular evidence linking GZMB downregulation to Treg functional impairment in this disease (51). In vitiligo, Tregs are often numerically reduced and functionally impaired; Chen et al. further demonstrated that patient Tregs undergo a phenotypic shift to a Th1-like T-bet-positive state with diminished ability to suppress CD8+ T cells, likely driven by the inflammatory milieu characteristic of the disease (52). A recent meta-analysis confirms reduced Treg counts, impaired suppressive capacity, and decreased IL-10 levels across vitiligo cohorts (53). Based on these observations, we hypothesize that the genetically mediated reduction of GZMB impairs Treg suppressive function in LO vitiligo patients, leading to insufficient restraint of autoreactive CD8+ T cells accumulated over time. This notion aligns with known age-related decreases in thymic Treg output and increased prevalence of inflammatory Treg phenotypes in older individuals. Thus, the observed circulating reduction of GZMB protein in LO patients likely reflects systemic Treg dysfunction rather than enhanced cytotoxicity. Although local NK and CD8+ T cell cytotoxic mechanisms cannot be excluded, our data and current literature suggest that granzyme B–dependent Treg impairment may represent a central mechanism in LO vitiligo pathogenesis. Therapeutic strategies aimed at restoring Treg granzyme B function, such as low-dose interleukin-2 or adoptive Treg transfer, could therefore be especially relevant for this population.

Among the prioritized targets, TEF demonstrates the clearest mechanistic alignment with the clinical validation findings and points toward potentially unappreciated connections between the circadian system and melanocyte vulnerability. Our data show that TEF mRNA and serum protein are significantly reduced exclusively in LO vitiligo patients, consistent with the SMR protective associations at both transcriptome and proteome levels. TEF is a member of the PAR bZIP transcription factor family and regulates circadian gene expression, xenobiotic detoxification, and metabolic homeostasis (54). In zebrafish embryonic cells, TEF has been shown to function as a master regulator of light-induced transcription, driving the expression of genes involved in DNA repair, oxidative stress response, and cell survival through PAR Response Elements in their promoters (55). Notably, TEF downregulation has also been observed in cutaneous melanoma transcriptome analyses, where disruptions of circadian genes including TEF were associated with altered immune cell infiltration patterns, suggesting that TEF may play a broader role in regulating the interface between melanocyte biology and immune surveillance (56). Based on these observations, we speculate that age-related decline in TEF expression may contribute to impaired circadian gating of cytoprotective programs in melanocytes, potentially rendering them more susceptible to oxidative damage over time; however, whether this mechanism directly underlies LO vitiligo susceptibility remains to be established (57). In immune cells, our single-cell analysis indicates that TEF is most highly expressed in CD4+ T cell subsets including memory Tregs, Th17 cells, and T follicular helper cells, raising the possibility that TEF downregulation may also affect circadian-dependent immune regulation in these populations, though this requires direct experimental validation. The age-related decline in circadian amplitude is well documented, and disrupted circadian rhythms in older individuals have been associated with heightened autoimmune risk (58, 59). TEF may therefore represent one molecular interface between age-associated circadian dysfunction and LO vitiligo timing, though this remains speculative. Future functional studies should examine whether TEF modulation can restore antioxidant gene expression and DNA repair capacity in aged melanocytes, and whether TEF governs circadian immune fluctuations relevant to T cell autoreactivity in LO disease.

The integration of spatial transcriptomic data provides unparalleled cellular resolution for genetic risk, laying a molecular foundation for potential therapeutically actionable insights. Our data-driven findings show that EO vitiligo genes are enriched in dermal mesenchymal cells and extracellular matrix-associated populations, suggesting a critical role for the skin microenvironment in early-age pathogenesis. Conversely, LO vitiligo genes localize predominantly to epidermal keratinocytes, which is consistent with the IFN-γ/CXCL10 axis where keratinocyte-derived chemokines orchestrate CD8+ T cell recruitment (60). Based on this distinct spatial separation and our broader genetic findings, we propose several forward-looking hypotheses for stratified medicine. Janus kinase (JAK) inhibitors—such as ruxolitinib, which achieved a 30% F-VASI75 response at week 24 and 52% at week 52 in phase 3 trials (8, 9)—primarily act on the interferon-gamma (IFN-γ) signaling pathway. Consequently, we hypothesize that these agents may be particularly effective in treating the epidermally localized, T cell-driven LO vitiligo. For EO patients, the mesenchymal and innate immune involvement suggests they might derive greater benefit from combined strategies targeting upstream innate immune mechanisms, melanocyte stress pathways, or emerging targets like IL-15 that sustain T and NK cell functions within the skin. We emphasize that while these stratified therapeutic implications are logically derived from our spatial and genetic data, they remain theoretical frameworks that necessitate direct validation in future clinical trials.

The genetic basis for vitiligo heterogeneity also informs understanding of comorbidity and protective effects. Compared to early-onset (EO) vitiligo, late-onset (LO) patients have a higher prevalence of autoimmune diseases, such as thyroid disorders and type 1 diabetes (61, 62). This is consistent with our observation of enriched adaptive immune responses and impaired regulatory T cell function in LO vitiligo, suggesting that immune dysregulation in this subtype may involve a more systemic breakdown of immune tolerance rather than a melanocyte-restricted process. Epidemiological data indicate that vitiligo patients experience a 38% to 61% decrease in melanoma incidence (63). However, whether this protective effect applies equally to EO and LO subtypes remains unclear. EO vitiligo, predominantly involving innate immune mechanisms, and LO vitiligo, characterized by CD8+ T cell–mediated adaptive immunity, might confer melanoma protection through fundamentally different immunological pathways. This distinction could be important when considering cancer risk assessments and patient counseling. Moreover, the genetic heterogeneity underlying these subtypes suggests that they may differ not only in disease mechanisms but also in clinical features and responses to therapy. Therefore, validating subtype-specific genetic risk variants in independent cohorts with detailed phenotyping, investigating functional roles of candidate genes such as TEF, examining differences in treatment efficacy, and developing genetic risk models incorporating subtype information will be key steps for translating these insights into precision medicine approaches tailored to patients’ individual disease profiles.

Several limitations should be acknowledged. First, our analyses relied on GWAS summary statistics rather than individual-level data, which constrained the refinement of subtype definitions. Second, the epidemiologically derived age-at-onset cutoffs may not fully capture the underlying biological heterogeneity compared to modeling onset age as a continuous variable. Third, a significant translational gap exists in our spatial mapping, as the gsMap framework utilized mouse embryonic data. While this approach identified that EO vitiligo genes are enriched in mesenchymal and extracellular matrix compartments, it is important to clarify that these findings reflect a specific developmental context. The direct extrapolation of these developmental signatures to adult human skin pathology requires further investigation using adult spatial datasets to confirm whether these tissue-specific patterns persist and contribute to disease mechanisms in mature skin. Furthermore, while our clinical validation aligns with the genetic findings, the modest sample size necessitates that these results be viewed as an exploratory validation. Larger independent cohorts and detailed functional studies remain essential to bridge these gaps and translate these insights into precision therapeutic strategies.

## Conclusion

In conclusion, this study demonstrates significant genetic heterogeneity between EO and LO vitiligo, identifying distinct risk genes, cellular mechanisms, and spatial localization patterns. These findings provide a foundation for precision medicine approaches that account for age-at-onset heterogeneity and may ultimately improve therapeutic outcomes for this common and often refractory condition.

## Data Availability

The data analyzed in this study are available from public repositories as follows. GWAS summary statistics for early-onset and late-onset vitiligo are deposited in the GWAS Catalog with accession numbers GCST007111 and GCST007112, respectively (https://www.ebi.ac.uk/gwas/). UK Biobank phenotype-specific GWAS datasets (UKB-PPP) can be obtained from https://www.synapse.org/#!Synapse:syn51364943 upon application. Cis-eQTL summary statistics were downloaded from the eQTLGen consortium website (https://www.eqtlgen.org/cis-eqtls.html). Single-cell transcriptomic data from the DICE database are available at https://dice-database.org/landing. These sources provide open access to all data underlying our analyses and support replication of the results.

## Glossary

ANOVA: Analysis of Variance
ATAC-seq: Assay for Transposase-Accessible Chromatin using sequencing
CD: Cluster of Differentiation
cDNA: Complementary Deoxyribonucleic Acid
DICE: Database of Immune Cell Expression
DNA: Deoxyribonucleic Acid
ELISA: Enzyme-Linked Immunosorbent Assay
EO: Early-Onset
eQTL: Expression Quantitative Trait Loci
F-VASI75: 75% Improvement in Facial Vitiligo Area Scoring Index
GAPDH: Glyceraldehyde-3-Phosphate Dehydrogenase
gsMap: Genetically informed Spatial Mapping of cells for complex traits
GSS: Gene-Spatial Score
GTEx: Genotype-Tissue Expression
GWAS: Genome-Wide Association Study
GZMB: Granzyme B
HEIDI: Heterogeneity in Dependent Instruments
HLA: Human Leukocyte Antigen
HRP: Horseradish Peroxidase
IFN-γ: Interferon-gamma
IL: Interleukin
JAK: Janus Kinase
KEGG: Kyoto Encyclopedia of Genes and Genomes
LD: Linkage Disequilibrium
LDSC: Linkage Disequilibrium Score Regression
LO: Late-Onset
MAGMA: Multi-marker Analysis of Genomic Annotation
MHC: Major Histocompatibility Complex
mRNA: Messenger Ribonucleic Acid
NK cell: Natural Killer cell
OD: Optical Density
OR: Odds Ratio
PBMC: Peripheral Blood Mononuclear Cell
PCC: Pearson Correlation Coefficient
PCR: Polymerase Chain Reaction
PGC: Primary Germ Cell
PPH: Posterior Probability of Hypothesis
pQTL: Protein Quantitative Trait Loci
qRT-PCR: Quantitative Reverse Transcription Polymerase Chain Reaction
RNA: Ribonucleic Acid
RNASET2: Ribonuclease T2
RNA-seq: Ribonucleic Acid Sequencing
SMR: Summary-data-based Mendelian Randomization
SNP: Single Nucleotide Polymorphism
TEF: Thyrotroph Embryonic Factor
Th cell: T helper cell
TMB: Tetramethylbenzidine
TPM: Transcripts Per Million
Treg: Regulatory T cell
UKB-PPP: UK Biobank Pharma Proteomics Project
